# DUDE-Seq: Fast, flexible, and robust denoising for targeted amplicon sequencing

**DOI:** 10.1371/journal.pone.0181463

**Published:** 2017-07-27

**Authors:** Byunghan Lee, Taesup Moon, Sungroh Yoon, Tsachy Weissman

**Affiliations:** 1 Electrical and Computer Engineering, Seoul National University, Seoul, Korea; 2 College of Information and Communication Engineering, Sungkyunkwan University, Suwon, Korea; 3 Interdisciplinary Program in Bioinformatics, Seoul National University, Seoul, Korea; 4 Neurology and Neurological Sciences, Stanford University, Stanford, California, United States of America; 5 Electrical Engineering, Stanford University, Stanford, California, United States of America; Mayo Clinic Arizona, UNITED STATES

## Abstract

We consider the correction of errors from nucleotide sequences produced by next-generation targeted amplicon sequencing. The next-generation sequencing (NGS) platforms can provide a great deal of sequencing data thanks to their high throughput, but the associated error rates often tend to be high. Denoising in high-throughput sequencing has thus become a crucial process for boosting the reliability of downstream analyses. Our methodology, named DUDE-Seq, is derived from a general setting of reconstructing finite-valued source data corrupted by a discrete memoryless channel and effectively corrects substitution and homopolymer indel errors, the two major types of sequencing errors in most high-throughput targeted amplicon sequencing platforms. Our experimental studies with real and simulated datasets suggest that the proposed DUDE-Seq not only outperforms existing alternatives in terms of error-correction capability and time efficiency, but also boosts the reliability of downstream analyses. Further, the flexibility of DUDE-Seq enables its robust application to different sequencing platforms and analysis pipelines by simple updates of the noise model. DUDE-Seq is available at http://data.snu.ac.kr/pub/dude-seq.

## Introduction

A new generation of high-throughput, low-cost sequencing technologies, referred to as *next-generation sequencing* (NGS) technologies [1], is reshaping biomedical research, including large-scale comparative and evolutionary studies [2–4]. Compared with automated Sanger sequencing, NGS platforms produce significantly shorter reads in large quantities, posing various new computational challenges [5].

There are several DNA sequencing methodologies that use NGS [6, 7] including whole genome sequencing (WGS), chromatin immunoprecipitation (ChIP) sequencing, and targeted sequencing. WGS is used to analyze the genome of an organism to capture all variants and identify potential causative variants; it is also used for *de novo* genome assembly. ChIP sequencing identifies genome-wide DNA binding sites for transcription factors and other proteins. Targeted sequencing (*e.g.*, exome sequencing and amplicon sequencing), the focus of this paper, is a cost-effective method that enables researchers to focus on investigating areas of interest that are likely to be involved in a particular phenotype. According to previous studies [8, 9], targeted sequencing often results in the complete coverage of exons of disease-related genes, while alternative methods result in approximately 90–95% coverage. Hence, in clinical settings, researchers tend to rely on targeted sequencing for diagnostic evaluations.

To detect sequences based on fluorescent labels at the molecular level, NGS technologies normally rely on imaging systems requiring templates that are amplified by emulsion polymerase chain reaction (PCR) or solid-phase amplification [1]. These amplification and imaging processes can generate erroneous reads, the origin of which can be traced to the incorrect determination of homopolymer lengths, the erroneous insertion/deletion/substitution of nucleotide bases, and PCR chimeras [6]. Substitution errors dominate for many platforms, including Illumina, while homopolymer errors, manifested as insertions and deletions (indels), are also abundant for 454 pyrosequencing and Ion Torrent.

Erroneous reads must be properly handled because they complicate downstream analyses (*e.g.*, variant calling and genome assembly), often lowering the quality of the whole analysis pipeline [7] Soft clipping, in which 3’-ends of a read are trimmed based on the quality scores of individual bases, may be the simplest approach, but it results in a loss of information [10]. More sophisticated methods focus on detecting and correcting errors in sequence data [11–20]. Given the widespread use of Illumina sequencing platforms, most error-correction algorithms have targeted substitution errors [10].

As summarized in recent reviews [10, 21], current error-correction methods for NGS data can be categorized as follows: *k*-mer (*i.e.*, oligonucleotides of length *k*) frequency/spectrum-based, multiple sequence alignment (MSA)-based, and statistical error model-based methods. The idea behind *k*-mer-based methods [13, 20, 22–25] is to create a list of “trusted” *k*-mers from the input reads and correct untrusted *k*-mers based on a consensus represented by this spectrum. In addition to the length of the *k*-mer, coverage (*k*-mer occurrences) information is important to determine trusted *k*-mers. Under the assumption that errors are rare and random and that coverage is uniform, for sufficiently large *k*, it is reasonable to expect that most errors alter *k*-mers to inexistent ones in a genome. Thus, for high-coverage genome sequences obtained by NGS, we may identify suspicious *k*-mers and correct them based on a consensus. MSA-based methods [12, 16, 26] work by aligning related sequences according to their similarities and correcting aligned reads, usually based on a consensus in an alignment column, using various techniques. This alignment-based scheme is inherently well-suited for correcting indel errors. Early methods suffered from computational issues, but recent approaches utilize advanced indexing techniques to expedite the alignments. In statistical error model-based methods [27–29], a statistical model is developed to capture the sequencing process, including error generation. In this regard, an empirical confusion model is often created from datasets, exploiting the information obtained from, *e.g.*, alignment results, Phred quality scores (a measure of the quality of nucleobases generated by automated DNA sequencing) [30], or other parameters.

While the above methods often exhibit good performance for various platforms, they also have several limitations. First, *k*-mer-based schemes tend to be ineligible when the coverage is expected to vary over the queried sequences, as in transcriptomics, metagenomics, heterogeneous cell samples, or pre-amplified libraries [21]. Second, MSA-based methods, which do not suffer from the above issue related to non-uniform coverage, often require the application of heuristic and sophisticated consensus decision rules for the aligned columns, and such rules may be sensitive to specific applications or sequencing platforms. Third, statistical error model-based methods typically use computationally expensive schemes (e.g., expectation-maximization) owing to additional stochastic modeling assumptions for the underlying DNA sequences. Moreover, little attention is given to the validity and accuracy of such modeling assumptions, let alone to theoretical analysis of whether near optimum or sound error-correction performance is attained. Finally, many existing schemes applying the three methods often return only representative (consensus) denoised sequences created by merging input sequences; hence, the number of sequences is often not preserved after denoising. In some applications, this may result in inconsistencies in downstream analyses. To address these limitations, many existing tools combine the three methods in a complementary manner to improve performance [10, 21].

In this paper, as an alternative, we applied an algorithm called Discrete Universal DEnoiser (DUDE) [31] for accurate DNA sequence denoising. DUDE was developed for a general setting of reconstructing sequences with finite-valued components (source symbols) corrupted by a noise mechanism that corrupts each source symbol independently and statistically identically. In the DNA denoising literature, such a noise model is equivalent to the confusion matrix commonly used in statistical error-model-based methods. As demonstrated in the original paper [31], DUDE exhibits rigorous performance guarantee for the following setting; even when no stochastic modeling assumptions are made for the underlying clean source data, only with the assumption of *known* noise mechanism, DUDE is shown to universally attain the optimum denoising performance for *any* source data the data increase. We note that the above setting of DUDE naturally fits the setting for DNA sequence denoising, *i.e.*, it is difficult to establish accurate stochastic models for clean DNA sequences, but it is simple and fairly realistic to assume noise models (*i.e.*, confusion matrices) for sequencing devices based on reference sequences.

The DUDE algorithm, which will be explained in details in the next section, possesses flavors that are somewhat connected to all three representative methods mentioned above, in a single scheme. Specifically, DUDE works with double-sided contexts of a fixed size that are analogous to *k*-mers. Moreover, like MSA, DUDE applies a denoising decision rule to each noisy symbol based on aggregated information over certain positions in the reads. However, unlike MSA, which makes a decision based on the information collected from the symbols in the same aligned column, DUDE makes a decision using the information collected from positions with the same double-sided context. Finally, the denoising decision rule of DUDE utilizes information from the assumed noise model, like in most statistical error model-based methods, but does not assume any stochastic model on the underlying sequence, thus resulting in a computationally efficient method. The method of incorporating the noise model is also simple, making it easy to flexibly apply DUDE to different sequencing platforms by simply changing the confusion matrix model in the algorithm.

With the above unique nature of the DUDE algorithm, we show in our experiments that it outperforms other state-of-the-art schemes, particularly for applications to targeted amplicon sequencing. Specifically, among the applicable areas of targeted amplicon sequencing (*e.g.*, cancer gene, 16S rRNA, plant, and animal sequencing [32]), we used 16S rRNA benchmark datasets obtained with different library preparation methods and DNA polymerases to confirm the robustness of our algorithm across various sequencing preparation methods. Targeted amplicon sequencing datasets often have deeper sequencing coverage than those of WGS or ChIP datasets, which frequently makes conventional *k*-mer-based techniques often suffer from the amplification bias problem [33]. By contrast, for DUDE-Seq, as the sequencing coverage becomes deeper, context-counting vectors can accumulate more probable contexts, and the robustness of denoising typically improves. We apply two versions of DUDE separately for substitution and homopolymer errors, the two major types of sequencing error. For substitution errors, our approach directly utilizes the original DUDE with appropriate adaptation to DNA sequences and is applicable to reads generated by any sequencing platform. For homopolymer errors, however, we do not apply the original DUDE, which was developed in a framework that does not cover errors of the homopolymer type. To correct homopolymer errors, we therefore adopt a variant of DUDE for general-output channels [34]. Our homopolymer-error correction is applicable to cases in which base-called sequences and the underlying flowgram intensities are available (*e.g.*, pyrosequencing and Ion Torrent). For brevity, we refer to both of these DUDE-based approaches as DUDE-Seq, but the correction type will be easily distinguishable by the reader.

## Discrete Universal DEnoiser (DUDE)

**Fig 1:**
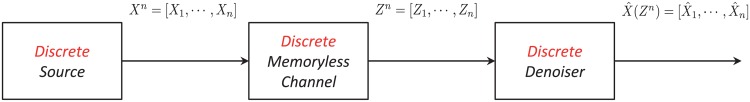
The general setting of discrete denoising.

In this section, we formally introduce the DUDE algorithm along with its notation and its connection to DNA sequence denoising. Fig 1 shows the concrete setting of the discrete denoising problem. We denote the underlying source data as {*x*_*i*_} and assume each component takes values in some finite set X. The resulting noisy version of the source corrupted by a noise mechanism is denoted as {*Z*_*i*_}, and its components take values in, again, some finite set Z. As mentioned in the Introduction, DUDE assumes that the noise mechanism injects noises that are independent and statistically identical, and such a mechanism is often referred to as a Discrete Memoryless Channel (DMC) in information theory. The DMC is completely characterized by the channel transition matrix, also known as the confusion matrix, $\Pi \in R^{\left|\text{X}|\times |\text{Z}\right|}$, of which the (*x*, *z*)-th element, Π(*x*, *z*), stands for Pr(*Z*_*i*_ = *z*|*x*_*i*_ = *x*), *i.e.*, the conditional probability that the noisy symbol takes value *z*, given that the original source symbol is *x*. We denote random variables with uppercase letters and the individual samples of random variables or deterministic symbols with lowercase letters. Thus, the underlying source data, which are treated by DUDE as individual sequences (and not a stochastic process), are denoted by the lowercase {*x*_*i*_}, and the noise-corrupted sequences, *i.e.*, sequences of random variables, are denoted by uppercase {*Z*_*i*_}. Furthermore, throughout this paper, we generally denote a sequence (*n*-tuple) as *a*^*n*^ = (*a*_1_,…,*a*_*n*_), for example, where $a_{i}^{j}$ refers to the subsequence (*a*_*i*_,…,*a*_*j*_).

As shown in Fig 1, a discrete denoiser observes the entire noisy data *Z*^*n*^ and reconstructs the original data with ${\hat{X}}^{n}=\left({\hat{X}}_{1}\left(Z^{n}\right),\ldots ,{\hat{X}}_{n}\left(Z^{n}\right)\right)$. The goodness of the reconstruction by a discrete denoiser ${\hat{X}}^{n}$ is measured by the average loss,

$$\begin{array}{c} L_{{\hat{X}}^{n}}\left(x^{n},Z^{n}\right)=\frac{1}{n}\sum_{i=1}^{n}\Lambda \left(x_{i},{\hat{X}}_{i}\left(Z^{n}\right)\right), \end{array}$$ (1)

where $\Lambda (x_{i},{\hat{x}}_{i})$ is a single-letter loss function that measures the loss incurred by estimating *x*_*i*_ with ${\hat{x}}_{i}$ at location *i*. The loss function can be also represented with a loss matrix $\Lambda \in R^{\left|\text{X}|\times \right|\hat{\text{X}}|}$.

DUDE in [31] is a two-pass algorithm that has linear complexity with respect to the data size *n*. During the first pass, given the realization of the noisy sequence *z*^*n*^, the algorithm collects the statistics vector

$$\begin{array}{c} m\left(z^{n},l^{k},r^{k}\right)\left[a\right]=|\left\{i:k+1\leq i\leq n-k,z_{i-k}^{i+k}=l^{k}ar^{k}\right\}|, \end{array}$$

for all a∈Z, which is the count of the occurrence of the symbol a∈Z along the noisy sequence *z*^*n*^ that has the *double-sided context* $\left(l^{k},r^{k}\right)\in {\text{Z}}^{2k}$. Note that **m** is similar to the counts across the aligned columns for the simple majority voting in MSA-based denoising methods. However, in DUDE, the count is collected regardless of whether the positions in the reads are aligned or not, but considering whether the position has the same context. Additionally, the context length *k* is analogous to the *k*-mer length. Once the **m** vector is collected, for the second pass, DUDE then applies the rule

$$\begin{array}{c} {\hat{X}}_{i}\left(z^{n}\right)=\arg\min_{\hat{x}\in \text{X}}m^{T}\left(z^{n},z_{i-k}^{i-1},z_{i+1}^{i+k}\right)\Pi^{-1}\left[\lambda_{\hat{x}}⊙\pi_{z_{i}}\right] \end{array}$$ (2)

for each *k* + 1 ≤ *i* ≤ *n* − *k*, where *π*_*z*_*i*__ is the *z*_*i*_-th column of the channel matrix **Π**, and $\lambda_{\hat{x}}$ is the $\hat{x}$-th column of the loss matrix **Λ**. Furthermore, ⊙ stands for the element-wise product operator for two vectors. The intuitive explanation of Eq (2) is as follows: when we rearrange the right-hand side of Eq (2), we obtain

$$\begin{array}{ccc} \left(2\right) & = & \arg\min_{\hat{x}\in \text{X}}\lambda_{\hat{x}}^{T}\{\pi_{z_{i}}⊙\Pi^{-T}m^{T}\left(z^{n},z_{i-k}^{i-1},z_{i+1}^{i+k}\right)\}, \end{array}$$ (3)

and we can show that *π*_*a*_ ⊙ **Π**^−*T*^ **m**^*T*^(*z*^*n*^, *l*^*k*^, *r*^*k*^) approximates the empirical count vector of the underlying *clean* symbol at the middle location that resulted in the noisy context *l*^*k*^*ar*^*k*^. Thus, the denoising rule Eq (2), re-expressed in Eq (3), finds a reconstruction symbol $\hat{x}$ that minimizes the expected loss with respect to the *empirical estimate* (obtained by utilizing the inverse of **Π**) of the count vector of the underlying *x*_*i*_ given the noisy context $z_{i-k}^{i+k}$. At a high level, DUDE is not a simple majority voting rule based on **m**; instead, it incorporates the DMC model **Π** (the confusion matrix) and loss function **Λ** to obtain a more accurate estimation of the clean source symbol. For more detailed and rigorous arguments on the intuitive description of Eq (2), we refer readers to the original paper [31, Section IV-B].

Note that formula Eq (2) assumes $\text{X}=\text{Z}=\hat{\text{X}}$ and **Π** is invertible for simplicity, but Weissman et al. [31] deal with more general cases as well. The form of Eq (2) also shows that DUDE is a sliding window denoiser with window size 2*k* + 1; *i.e.*, DUDE returns the same denoised symbol at all locations with the same value $z_{i-k}^{i+k}$. DUDE is guaranteed to attain the optimum performance by the sliding window denoisers with the same window size as the observation length *n* increases. For more details on the theoretical performance analyses, see Weissman et al. [31, Section V].

The original DUDE dealt exclusively with the case of |X| and |Z| finite. Dembo and Weissman [34] DUDE to the case of discrete input and general output channels; the noisy outputs do not have to have their values in some finite set, but can have continuous values as well. As in [31], the memoryless noisy channel model, which is characterized in this case by the set of densities ${\left\{f_{x}\right\}}_{x\in \text{X}}$, was assumed to be known. As shown in [34, Fig 1], the crux of the arguments is to apply a scalar quantizer *Q*(⋅) to each continuous-valued noisy output {*Y*_*i*_} and to derive a virtual DMC, $\Gamma \in R^{\left|\text{X}|\times |\text{Z}\right|}$, between the discrete input {*X*_*i*_} and the quantized (hence, discrete) output {*Z*_*i*_}. Such **Γ** can be readily obtained by the knowledge of ${\left\{f_{x}\right\}}_{x\in \text{X}}$ and evaluating the following integral for each (*x*, *z*): Γ(*x*, *z*) = ∫_*y*:*Q*(*y*) = *z*_ *f*_*x*_(*y*)*dy*. Once the virtual DMC is obtained, the rest of the algorithm in [34] proceeds similarly as the original DUDE; specifically, it obtains the statistics vector **m** for the quantized noisy outputs {*Z*_*i*_} during the first pass, and then applies a sliding window denoising rule similar to Eq (2), which depends on the statistics vector **m**, the virtual DMC **Γ**, ${\left\{f_{x}\right\}}_{x\in \text{X}}$, and the noisy sequence *Y*^*n*^, during the second pass. A concrete denoising rule can be found in [34, Eqs (16), (19) and (20)]. In [34], a formal analysis of the generalized DUDE shows that it attains the optimum denoising performance among sliding window denoisers with the same window size, that base their denoising decisions on the original continuous-valued outputs *Y*^*n*^. We refer readers to the paper for more details. In the next section, we show how we adopt this generalized DUDE in our DUDE-Seq to correct homopolymer errors in DNA sequencing.

## DUDE-Seq: DUDE for DNA sequence denoising

### Substitution errors

**Fig 2:**
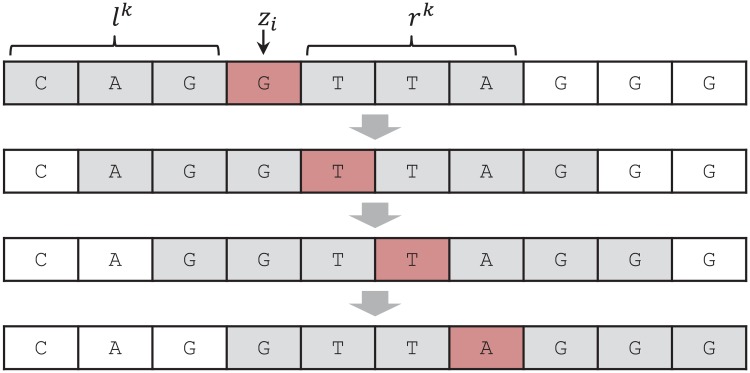
A sliding window procedure of the DUDE-Seq with the context size k = 3. During the first pass, DUDE-Seq updates the **m**(*z*^*n*^, *l*^3^, *r*^3^) for the encountered double-sided contexts (*l*^3^, *r*^3^). Then, for the second pass, DUDE-Seq uses the obtained **m**(*z*^*n*^, *l*^3^, *r*^3^) and Eq (2) for the denoising.

As described in the previous section, the setting of the original DUDE algorithm naturally aligns with the setting of substitution-error correction in DNA sequence denoising. We can set X=Z={A,C,G,T}, and the loss function as the Hamming loss, namely, $\Lambda (x,\hat{x})=0$, if $x=\hat{x}$, and $\Lambda (x,\hat{x})=1$, otherwise. Then, the two-pass sliding window procedure of DUDE for collecting the statistics vector **m** and the actual denoising can be directly applied as shown in the toy example in Fig 2. Before we formally describe our DUDE-Seq for substitution-error correction, however, we need to address some subtle points.

First, the original DUDE in Eq (2) assumes that the DMC matrix **Π** is known beforehand, but in real DNA sequence denoising, we need to estimate **Π** for each sequencing device. As described in the Experimental Results section in detail, we performed this estimation following the typical process for obtaining the empirical confusion matrix, *i.e.*, we aligned the predefined reference sequence and its noise-corrupted sequence and then determined the ratio of substitution errors and obtain the estimated **Π**. Second, the original DUDE assumes that the noise mechanism is memoryless, *i.e.*, the error rate does not depend on the location of a base within the sequence. In contrast, for real sequencing devices, the actual error rate, namely, the conditional probability Pr(*Z*_*i*_ = *z*|*X*_*i*_ = *x*) may not always be the same for all location index values *i*. For example, for Illumina sequencers, the error rate tends to increase towards the ends of reads, as pointed out in [21]. In our DUDE-Seq, however, we still treat the substitution error mechanism as a DMC and therefore use the single estimated **Π** obtained as above, which is essentially the same as that obtained using the *average* error rate matrix. Our experimental results show that such an approach still yields very competitive denoising results. Thirdly, the optimality of the original DUDE relies on the stationarity of the underlying clean sequence, thus requiring a very large observation sequence length *n* to obtain a reliable statistics vector **m**. In contrast, most sequencing devices generate multiple short reads of lengths 100 ∼ 200. Hence, in DUDE-Seq, we combined all statistics vectors collected from multiple short reads to generate a single statistics vector **m** to use in Eq (2).

Addressing the above three points, a formal summary of DUDE-Seq for the substitution errors is given in Algorithm 1. Note that the pseudocode in Algorithm 1 skips those bases whose Phred quality score s are higher than a user-specified threshold and invokes DUDE-Seq only for the bases with low quality scores (lines 10–14). This is in accord with the common practice in sequence preprocessing and is not a specific property of the DUDE-Seq algorithm. Furthermore, for simplicity, we denoted *z*^*n*^ as the entire noisy DNA sequence, and $m^{T}\left(z^{n},z_{i-k}^{i-1},z_{i+1}^{i+k}\right)$ represents the aggregated statistics vector obtained as described above.

**Algorithm 1** The *DUDE-Seq* for substitution errors

**Require:** Observation *z*^*n*^, Estimated DMC matrix $\Pi \in R^{4\times 4}$, Hamming loss $\Lambda \in R^{4\times 4}$, Context size *k*, Phred quality score *Q*^*n*^

**Ensure:** The denoised sequence ${\hat{X}}^{n}$

1: Define $m\left(z^{n},l^{k},r^{k}\right)\in R^{4}$ for all (*l*^*k*^, *r*^*k*^)∈{A,C,G,T}^2*k*^.

2: Initialize **m**(*z*^*n*^, *l*^*k*^, *r*^*k*^)[*a*] = 0 for all (*l*^*k*^, *r*^*k*^)∈{A,C,G,T}^2*k*^ and for all *a* ∈ {A,C,G,T}

3: **For** *i* ← *k* + 1,…, *n* − *k* **do**                  ⊳ First pass

4:  $m\left(z^{n},z_{i-k}^{i-1},z_{i+1}^{i+k}\right)\left[z_{i}\right]=m\left(z^{n},z_{i-k}^{i-1},z_{i+1}^{i+k}\right)\left[z_{i}\right]+1$ ⊳ Update the count statistics vector

5: **end for**

6: **for** *i* ← 1,…, *n* **do**                    ⊳ Second pass

7:  **if** *i* ≤ *k* or *i* ≥ *n* − *k* + 1 **then**

8:   ${\hat{X}}_{i}=z_{i}$

9:  **else**

10:   **if** *Q*_*i*_ > threshold **then**                ⊳ Quality score

11:    ${\hat{X}}_{i}=z_{i}$

12:   **else**

13:    ${\hat{X}}_{i}\left(z^{n}\right)=\underset{\hat{x}\in \left\{A,C,G,T\right\}}{\mathrm{argmin}}m^{T}\left(z^{n},z_{i-k}^{i-1},z_{i+1}^{i+k}\right)\Pi^{-1}\left[\lambda_{\hat{x}}⊙\pi_{z_{i}}\right]$  ⊳ Apply the denoising rule

14:   **end if**

15:  **end if**

16: **end for**

#### Remarks

1. Incorporating flanking sequences in DUDE-Seq is quite straightforward; we can simply use the one-sided contexts *l*^2*k*^ or *r*^2*k*^ once DUDE-Seq reaches the flanking regions. In our experiments, however, we did not perform such modification (lines 7–8 of Algorithm 1) since we normally used small *k* values (around *k* = 5). As demonstrated in our experimental results, the effect of such small flanking regions is not significant on the final denoising results, and we can achieve satisfactory results without considering flanking regions. However, in general, should longer values of *k* be needed, we can easily modify the algorithm to incorporate one-sided contexts in the flanking regions, and such modification will clearly improve the final denoising result.
2. DUDE-Seq does not need to consider reverse complements of the input sequences to collect **m**’s, since forward and reverse reads are handled separately in our experiments. Reverse complements are typically considered when we need to handle double-stranded sequences without knowing whether each read corresponds to the forward or reverse strand.

### Homopolymer errors

**Fig 3:**
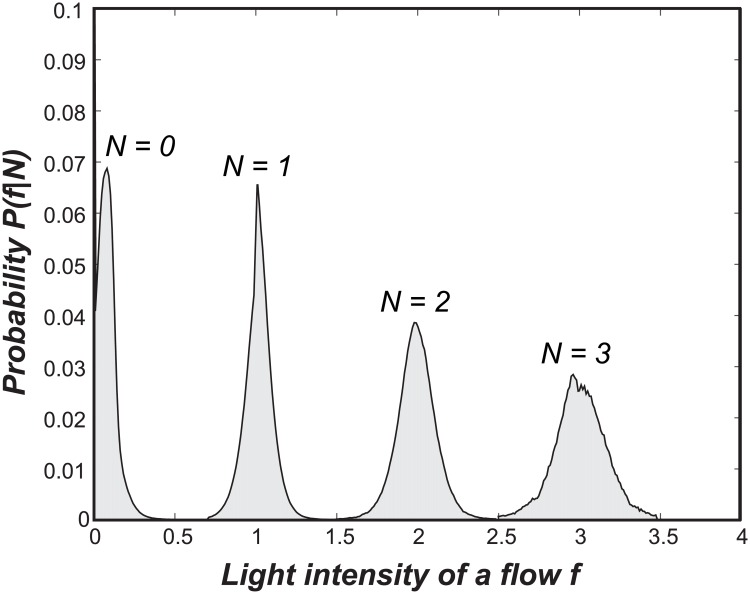
Conditional intensity distributions for *N* = 0, 1, 2, 3.

Homopolymer errors, particularly in pyrosequencing, occur while handling the observed flowgram, and a careful understanding of the error injection procedure is necessary to correct these errors. As described in [35], in pyrosequencing, the light intensities, *i.e.*, flowgram, that correspond to a fixed order of four DNA bases {T, A, C, G} are sequentially observed. The intensity value increases when the number of consecutive nucleotides (*i.e.*, homopolymers) for each DNA base increases, and the standard base-calling procedure rounds the continuous-valued intensities to the closest integers. For example, when the observed light intensities for the two frames of DNA bases are [0.03 1.03 0.09 0.12; 1.89 0.09 0.09 1.01], the corresponding rounded integers are [0.00 1.00 0.00 0.00; 2.00 0.00 0.00 1.00]. Hence, the resulting sequence is ATTG. The insertion and deletion errors are inferred because the observed light intensities do not perfectly match the actual homopolymer lengths; thus, the rounding procedure may result in the insertion or deletion of DNA symbols. In fact, the distribution of the intensities *f*, given the actual homopolymer length *N*, {*P*(*f*|*N*)}, can be obtained for each sequencing device, and Fig 3 shows typical distributions given various lengths.

Exploiting the fact that the order of DNA bases is always fixed at {T, A, C, G}, we can apply the setting of the generalized DUDE in [34] to correct homopolymer errors as follows. Because we know the exact DNA base that corresponds with each intensity value, the goal is the correct estimatimation of homopolymer lengths from the observed intensity values. Hence, we can interpret the intensity distributions {*P*(*f*|*N*)} as the memoryless noisy channel models with a continuous-output, where the channel input is the homopolymer length *N*. We set the upper bound of *N* to 9 according to the convention commonly used for handling flowgram distributions in the targeted amplicon sequencing literature [35–37]. When the usual rounding function

$$\begin{array}{c} Q_{R}\left(f\right)=\underset{i\in \{0,\ldots ,9\}}{\mathrm{argmin}}\;|i-f| \end{array}$$ (4)

is used as a scalar quantizer, as mentioned above, and the virtual DMC $\Gamma \in R^{10\times 10}$ can be obtained by calculating the integral

$$\begin{array}{c} \Gamma \left(i,j\right)=\int_{j-0.5}^{j+0.5}\;P\left(f|i\right)df \end{array}$$ (5)

for each 0 ≤ *i* ≤ 9, 1 ≤ *j* ≤ 9 and $\Gamma \left(i,0\right)=\int_{0}^{0.5}P\left(f|i\right)df$.

With this virtual DMC model, we apply a scheme inspired by the generalized DUDE to correctly estimate the homopolymer lengths, which results in correcting the insertion and deletion errors. That is, we set X=Z={0,1,…,9}, and again use the Hamming loss $\Lambda \in R^{10\times 10}$. With this setting, we apply *Q*_*R*_(*f*) to each *f*_*i*_ to obtain the quantized discrete output *z*_*i*_, and obtain the count statistics vector **m** from *z*^*n*^ during the first pass. Then, for the second pass, instead of applying the more involved denoising rule in [34, we employ the same rule as Eq (2) with **Γ** in place of **Π** to obtain the denoised sequence of integers ${\hat{X}}^{n}$ based on the quantized noisy sequence *Z^n^*. Although it is its implementation is easier and it has a faster running time than that of the generalized DUDE. Once we obtain ${\hat{X}}^{n}$, from the knowledge of the DNA base for each *i*, we can reconstruct the homopolymer error-corrected DNA sequence $\hat{D}$ (the length of which may not necessarily be equal to *n*). Algorithm 2 summarizes the pseudo-code of DUDE-Seq for homopolymer-error correction.

## Experimental results

### Setup

We used both real and simulated NGS datasets and compared the performance of DUDE-Seq with that of several state-of-the-art error correction methods. The list of alternative tools used for comparison and the rationale behind our choice s are described in the next subsection. When the flowgram intensities of base-calling were available, we corrected both homopolymer and substitution errors; otherwise, we only corrected substitution errors. The specifications of the machine we used for the analysis are as follows: Ubuntu 12.04.3 LTS, 2 × Intel Xeon X5650 CPUs, 64 GB main memory, and 2 TB HDD.

**Algorithm 2** The *DUDE-Seq* for homopolymer errors

**Require:** Flowgram data *f*^*n*^, Flowgram densities ${\left\{P\left(f|N\right)\right\}}_{N=0}^{9}$, Hamming loss $\Lambda \in R^{10\times 10}$, Context size *k*

**Ensure:** The denoised sequence $\hat{D}$

1: Let *Q*_*R*_(*f*) be the rounding quantizer in Eq (4) of the main text

2: Let Base(*i*) ∈ {T, A, C, G} be the DNA base corresponding to *f*_*i*_

3: Define $m\left(f^{n},l^{k},r^{k}\right)\in R^{10}$ for all (*l*^*k*^, *r*^*k*^) ∈ {0, 1,…,9}^2*k*^.

4: Initialize **m**(*f*^*n*^, *l*^*k*^, *r*^*k*^)[*a*] = 0 for all (*l*^*k*^, *r*^*k*^) ∈ {0, 1,…,9}^2*k*^ and for all *a* ∈ {0, 1,…,9}

5: Let $\hat{D}=\phi$, *I* = 0

6: **for** *i* ← 0,…,9 **do**

7:  **for** *j* ← 0,…,9 **do**

8:   Compute Γ(*i*, *j*) following Eq (5) of the main text  ⊳ Computing the virtual DMC **Γ**

9:  **end for**

10: **end for**

11: **for** *i* ← 1,…,*n* **do** Obtain *z*_*i*_ = *Q*_*R*_(*f*_*i*_)           ⊳ Note *z*_*i*_ ∈ {0,…,9}

12: **end for**

13: **for** *i* ← *k* + 1,…,*n* − *k* **do**                  ⊳ First pass

14:  $m\left(f^{n},z_{i-k}^{i-1},z_{i+1}^{i+k}\right)\left[z_{i}\right]=m\left(f^{n},z_{i-k}^{i-1},z_{i+1}^{i+k}\right)\left[z_{i}\right]+1$

15: **end for**

16: **for** *i* ← 1,…,*n* **do**                    ⊳ Second pass

17:  **if** *i* ≤ *k* or *i* ≥ *n* − *k* + 1 **then** ${\hat{X}}_{i}\left(f^{n}\right)=z_{i}$

18:  **else**

19:   ${\hat{X}}_{i}\left(f^{n}\right)=\underset{\hat{x}\in \text{X}}{\mathrm{argmin}}m^{T}\left(f^{n},z_{i-k}^{i-1},z_{i+1}^{i+k}\right)\Gamma^{-1}\left[\lambda_{\hat{x}}⊙\gamma_{z_{i}}\right]$ ⊳ Note ${\hat{X}}_{i}\left(z^{n}\right)\in \left\{0,\ldots ,9\right\}$

20:  **end if**

21:  **if** ${\hat{X}}_{i}\left(f^{n}\right)\geq 1$ **then**

22:   **for** $j\leftarrow 1,\ldots ,{\hat{X}}_{i}\left(f^{n}\right)$ **do** ${\hat{D}}_{I+j}=\text{Base}\left(i\right)$ ⊳ Reconstructing the DNA sequence

23:   **end for**

24:  **end if**

25:  $I\leftarrow I+{\hat{X}}_{i}\left(f^{n}\right)$

26: **end for**

**Fig 4:**
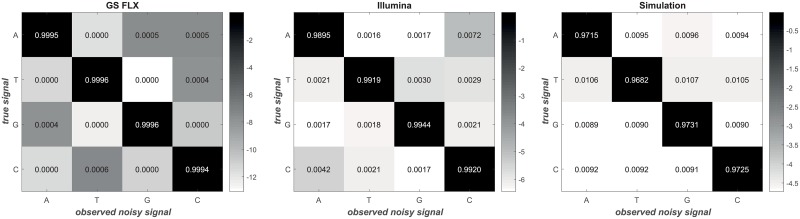
Adjustable DMC matrix Π of DUDE-Seq. Empirically obtained **Π**’s for different sequencing platforms (colors are on a log scale).

DUDE-Seq has a single hyperparameter *k*, the context size, that needs to be determined. Similar to the popular *k*-mer-based schemes, there is no analytical method for selecting the best *k* for finite data size *n*, except for the asymptotic order result of ${k|\text{X}|}^{2k}=o\left(n/\log n\right)$ in [31], but a heuristic rule of thumb is to try values between 2 and 8. Furthermore, as shown in Eq (2), the two adjustable matrices, **Λ** and **Π**, are required for DUDE-Seq. The loss **Λ** used for both types of errors is the Hamming loss. According to Marinier *et al.* [38], adjusting the sequence length by one can correct most homopolymer errors, which justifies our use of Hamming loss in DUDE-Seq. In our experiments, the use of other types of loss functions did not result in any noticeable performance differences. The DMC matrix Π for substitution errors is empirically determined by aligning each sampled read to its reference sequence, as in [35]. Fig 4 shows the non-negligible variation in the empirically obtained **Π**’s across the sequencing platforms, where each row corresponds to the true signal *x* and each column corresponds to the observed noisy signal *z*. In this setting, each cell represents the conditional probability *P*(*z*|*x*). In our experiments, dataset P1–P8 used **Π** for GS FLX, Q19–Q31 used **Π** for Illumina, and S5, A5 used **Π** for Simulation data. The details of each dataset are explained in the following sections.

In order to evaluate the results, we used Burrows-Wheeler Aligner (BWA) [39] and SAMtools [40]. We aligned all reads to their reference genome using BWA with the following parameters: [minimum seed length: 19, matching score: 1, mismatch penalty: 4, gap open penalty: 6, gap extension penalty: 1]. After the mapped regions were determined using BWA in SAM format, we chose uniquely mapped pairs using SAMtools. The Compact Idiosyncratic Gapped Alignment Report (CIGAR) string and MD tag (string for mismatching positions) for each of the resultant pairs in the SAM file were reconstructed to their pairwise alignments using sam2pairwise [41].

### Evaluation metric

As a performance measure, we define the per-base error rate of a tool after denoising as

$$e_{\text{tool}}=\frac{\#\text{mismatched bases}}{\#\text{aligned bases}},$$ (6)

in which ‘# aligned bases’ represents the number of mapped bases (*i.e.*, matches and mismatches) after mapping each read to its reference sequence, and ‘# mismatched bases’ represents the number of the erroneous bases (*i.e.*, insertions, deletions, and substitutions) among the aligned bases.

We also employ an alternative definition that adjusts the error rate by incorporating the degree of alignment. To this end, we define the *relative gain* of the number of aligned bases after denoising by a tool over raw data as

$$\begin{array}{c} g\left(a_{\text{tool}}\right)=\frac{\#\text{aligned bases after denoising}-\#\text{aligned bases in raw}}{\#\text{aligned bases in raw}}. \end{array}$$ (7)

Based on this, the adjusted error rate ${\hat{e}}_{\text{tool}}$ of a denoising tool is defined as follows:

$$\begin{array}{c} {\hat{e}}_{\text{tool}}=\left(1+g\left(a_{\text{tool}}\right)\right)\times e_{\text{tool}}-g\left(a_{\text{tool}}\right)\times e_{\text{raw}}, \end{array}$$ (8)

where *e*_tool_ and *e*_raw_ represent the (unadjusted) error rates of the denoised data and the raw data, respectively. In other words, Eq (8) is a weighted average of *e*_tool_ and *e*_raw_, in which the weights are determined by the relative number of aligned bases of a tool compared to the raw sequence. We believe ${\hat{e}}_{\text{tool}}$ is a fairer measure as it penalizes the error rate of a denoiser when there is a small number of aligned bases. The relative gain of the adjusted error rate over raw data is then defined as

$$\begin{array}{c} g\left({\hat{e}}_{\text{tool}}\right)=\frac{e_{\text{raw}}-{\hat{e}}_{\text{tool}}}{e_{\text{raw}}}, \end{array}$$ (9)

which we use to evaluate the denoiser performance.

While evaluating a clustering result, we employ a measure of concordance (MoC) [42] which is a popular similarity measure for pairs of clusterings. For two pairs of clusterings *P* and *Q* with *I* and *J* clusters, respectively, the MoC is defined as

$$\text{MoC}(P,Q)=\frac{1}{\sqrt{IJ}-1}\left(\sum_{i=1}^{I}\sum_{j=1}^{J}\frac{f_{ij}^{2}}{p_{i}q_{j}}-1\right)$$ (10)

where *f_ij_* is the number of the common objects between cluster *P_i_* and *Q_j_* when *p_i_* and *q_j_* are the numbers of the objects in cluster *P_i_* and *Q_j_*, respectively. A MoC of one or zero represents perfect or no concordance, respectively, between the two clusters.

### Software chosen for comparison

It is impossible to compare the performance of DUDE-Seq with that of all other schemes. Hence, we selected representative baselines using the following reasoning.

1. We included tools that can represent different principles outlined in the Introduction, namely, *k*-mer-based (Trowel, Reptile, BLESS, and fermi), MSA-based (Coral), and statistical error model-based (AmpliconNoise) methods.
2. We considered the recommendations of [21] to choose baseline tools that are competitive for different scenarios, *i.e.*, for 454 pyrosequencing data (AmpliconNoise), non-uniform coverage data, such as metagenomics data (Trowel, fermi, Reptile), data dominated by substitution errors, such as Illumina data (Trowel, fermi, Reptile), and data with a high prevalence of indel errors (Coral).
3. For multiple *k*-mer-based tools, we chose those that use different main approaches/data structures: BLESS (*k*-mer spectrum-based/hash table and bloom filter), fermi (*k*-mer spectrum and frequency-based/hash table and suffix array), Trowel (*k*-mer spectrum-based/hash table), and Reptile (*k*-mer frequency and Hamming graph-based/replicated sorted *k*-mer list).
4. The selected tools were developed quite recently; Trowel and BLESS (2014), fermi (2012), Coral and AmpliconNoise (2011), and Reptile (2010).
5. We mainly chose tools that return read-by-read denoising results to make fair error-rate comparisons with DUDE-seq. We excluded tools that return a substantially reduced number of reads after error correction (caused by filtering or forming consensus clusters). Examples of excluded tools are Acacia, ALLPATHS-LG, and SOAPdenovo.
6. We also excluded some recently developed tools that require additional mandatory information (e.g., the size of the genome of the reference organism) beyond the common setting of DNA sequence denoising in order to make fair error-rate comparisons. Examples of excluded tools are Fiona, Blue, and Lighter. Incorporating those tools that require additional information into the DUDE-Seq framework and comparisons with the excluded tools would be another future directions.

### Real data: 454 pyrosequencing

Pyrosequenced 16S rRNA genes are commonly used to characterize microbial communities because the method yields relatively longer reads than those of other NGS technologies [43]. Although 454 pyrosequencing is gradually being phased out, we test ed DUDE-Seq with 454 pyrosequencing data for the following reasons: (1) the DUDE-Seq methodology for correcting homopolymeric errors in 454 sequencing data is equally applicable to other sequencing technologies that produce homopolymeric errors, such as Ion Torrent; (2) using pyrosequencing data allows us to exploit existing (experimentally obtained) estimates of the channel transition matrix **Γ** (*e.g.*, [35]), which is required for denoising noisy flowgrams by DUDE-Seq (see Algorithm 2); (3) in the metagenomics literature, widely used standard benchmarks consist of datasets generated by pyrosequencing.

In metagenome analysis [44], grouping reads and assigning them to operational taxonomic units (OTUs) (*i.e.*, binning) are essential processes, given that the majority of microbial species have not been taxonomically classified. By OTU binning, we can computationally identify closely related genetic groups of reads at a desired level of sequence differences. However, owing to erroneous reads, nonexistent OTUs may be obtained, resulting in the common problem of overestimating ground truth OTUs. Such overestimation is a bottleneck in the overall microbiome analysis; hence, removing errors in reads before they are assigned to OTUs is a critical issue [35]. With this motivation, in some of our experiments below, we used the difference between the number of assigned OTUs and the ground truth number of OTUs as a proxy for denoising performance; the number of OTUs was determined using UCLUST [45] at identity threshold of 0.97 which is for species assignment.

We tested the performance of DUDE-Seq with the eight datasets used in [35], which are mixtures of 94 environmental clones library from eutrophic lake (Priest Pot) using primers 787f and 1492r. Dataset P1 had 90 clones that are mixed in two orders of magnitude difference while P2 had 23 clones that were mixed in equal proportions. In P3, P4, and P5 and P6, P7, and P8, there are 87 mock communities mixed in even and uneven proportions, respectively. In all datasets, both homopolymer and substitution errors exist, and the flowgram intensity values as well as the distributions are available [35]. Therefore, DUDE-Seq tries to correct both types of errors using the empirically obtained **Π** and the flowgram intensity distributions {*P*(*f*|*N*)}.

**Fig 5:**
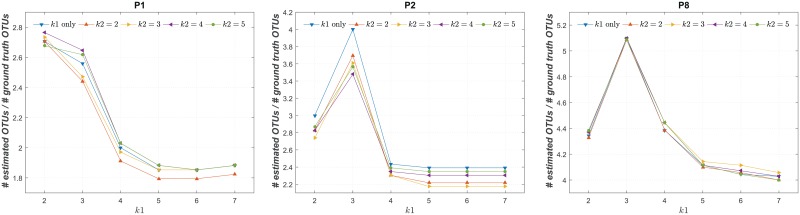
Hyperparameter *k* of DUDE-Seq. Effects of varying context size *k* [*k*1 is for Algorithm 1 (substitution-error correction) and *k*2 is for Algorithm 2 (homopolymer-error correction); data: [35]].

We first show the effect of *k* on the performance of DUDE-Seq in Fig 5. The vertical axis shows the ratio between the number of OTUs assigned after denoising with DUDE-Seq and the ground truth number of OTUs for the P1, P2, and P8 dataset. The horizontal axis shows the *k* values used for correcting the substitution errors (*i.e.*, for Algorithm 1), and color-coded curves were generated for different *k* values used for homopolymer-error correction (*i.e.*, for Algorithm 2). As shown in the figure, correcting homopolymer errors (*i.e.*, with *k* = 2 for Algorithm 2) always enhanceed the results in terms of the number of OTUs in comparison to correcting substitution errors alone (*i.e.*, Algorithm 1 alone). We observe that *k* = 5 for Algorithm 1 and *k* = 2 for Algorithm 2 produce the best results in terms of the number of OTUs. Larger *k* value work better for substitution errors owing to the smaller alphabet size of the data, *i.e.*, 4, compared to that of homopolymer errors, *i.e.*, 10. Motivated by this result, we fixed the context sizes of substitution error correction and homopolymer error correction to *k* = 5 and *k* = 2, respectively, for all subsequent experiments.

**Fig 6:**
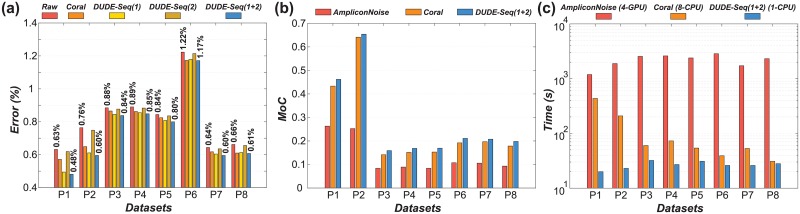
Comparison of reads correction performance on eight real 454 pyrosequencing datasets (labeled P1–P8; [35]). [parameters: *k* = 5 (Algorithm 1) and *k* = 2 (Algorithm 2) for DUDE-Seq; (*s*_PyroNoise_, *c*_PyroNoise_, *s*_SeqNoise_, *c*_SeqNoise_) = (60, 0.01, 25, 0.08) for AmpliconNoise; (*k*, *mr*, *mm*, *g*) = (21, 2, 2, 3) for Coral]: (a) Per-base error rates [1 and 2 represents substitution error-correction (Algorithm 1) and homopolymer error-correction (Algorithm 2), respectively.] (b) Measure of concordance (MoC), a similarity measure for pairs of clusterings (c) Running time (the type and quantity of processors used for each case are shown in legend).

In Fig 6(a), we report a more direct analysis of error correction performance. We compared the performance of DUDE-Seq with that of Coral [16], which is an MSA-based state-of-the-art scheme. It aligns multiple reads by exploiting the *k*-mer neighborhood of each base read and produces read-by-read correction results for pyrosequencing datasets, similar to DUDE-Seq. Furthermore, as a baseline, we also present ed the error rates for the original, uncorrected sequences (labeled ‘Raw’). We did not include the results of AmpliconNoise [35], a state-of-the-art scheme for 454 pyrosequencing data, in the performance comparison because it does not provide read-by-read correction results, making a fair comparison of the per-base error correction performance with DUDE-Seq difficult. We observeed that DUDE-Seq(1+2), which corrects both substitution errors and homopolymer errors, always outperforms Coral, and the relative error reductions of DUDE-Seq(1+2) with respect to ‘Raw,’ without any denoising, was up to 23.8%. Furthermore, the homopolymer error correction further drives down the error rates obtained by substitution-error correction alone; hence, DUDE-Seq(1+2) always outperforms DUDE-Seq(1).

In Fig 6(b), we compare the error correction performance of three schemes, AmpliconNoise, Coral, and DUDE-Seq, in terms of the MoC. AmpliconNoise assumes a certain statistical model on the DNA sequence and runs an expectation-maximization algorithm for denoising. Here, the two clusterings in the comparison are the golden OTU clusterings and the clusterings returned by denoisers. We observe that for all eight datasets, the number of OTUs generated by DUDE-Seq is consistently closer to the ground truth, providing higher MoC values than those of the other two schemes.

Furthermore, Fig 6(c) compares the running time of the three schemes for the eight datasets. We can clearly see that DUDE-Seq is substantially faster than the other two. Particularly, we stress that the running time of DUDE-Seq, even when implemented and executed with a single CPU, is two orders of magnitude faster than that of parallelized AmpliconNoise, run on four powerful GPUs. We believe that this substantial boost over state-of-the-art schemes with respect to running time is a compelling reason for the adoption of DUDE-Seq in microbial community analysis.

### Real data: Illumina sequencing

Illumina platforms, such as GAIIx, MiSeq, and HiSeq, are currently ubiquitous platforms in genome analysis. These platforms intrinsically generate paired-end reads (forward and reverse reads), due to the relatively short reads compared to those obtained by automated Sanger sequencing [46]. Merging the forward and reverse reads from paired-end sequencing yeilds elongated reads (*e.g.*, 2 × 300 bp for MiSeq) that improve the performance of downstream pipelines [47].

Illumina platforms primarily inject substitution errors. A realistic error model is not the DMC, though, as the error rates of the Illumina tend to increase from the beginning to the end of reads. Thus, the assumptions under which the DUDE was originally developed do not exactly apply to the error model of Illumina. In our experiments with DUDE-Seq, however, we still used the empirically obtained DMC model **Π** in Fig 4, which was computed by *averaging* all error rates throughout different Illumina platforms.

**Fig 7:**
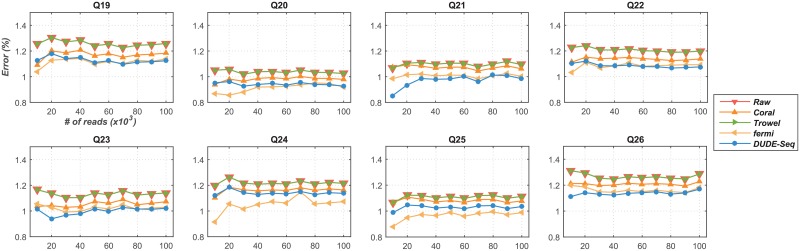
Comparison of reads correction performance on real Illumina datasets (labeled Q19–Q26; see Table 1 for more details). [parameters: (*k*, *mr*, *mm*, *g*) = (21, 1, 1, 1000) for Coral; *k* = 21 for Trowel; (*k*, *O*, *C*, *s*) = (21, 3, 0.3, 5) for fermi; *k* = 5 for DUDE-Seq; no BLESS result shown since it did not work on these data] [Organisms: *Anaerocellum thermophilum Z-1320 DSM 6725* (Q19 and Q23), *Bacteroides thetaiotaomicron VPI-5482* (Q20 and Q24), *Bacteroides vulgatus ATCC 8482* (Q21 and Q25), *Caldicellulosiruptor saccharolyticus DSM 8903* (Q22 and Q26)] [Q19–Q22: Miseq (Library: nested single index, Taq: Q5 neb, Primer: 515 & 805RA)] [Q23–Q26: Miseq (Library: NexteraXT, Taq: Q5 neb, Primer: 341f & 806rcb)].

In our experiments, we used 13 real Illumina datasets (named Q19–Q31) reported previously [32], including sequencing results from four organisms (*Anaerocellum thermophilum Z-1320 DSM 6725*, *Bacteroides thetaiotaomicron VPI-5482*, *Bacteroides vulgatus ATCC 8482*, and *Caldicellulosiruptor saccharolyticus DSM 8903*) targeting two hypervariable regions, V3 and V4, using different configurations (see the caption for Table 1 and Fig 7 for details). To examine how the number of reads in a dataset affects denoising performance, we derived 10 subsets from the original datasets by randomly subsampling 10,000 to 100,000 reads in increments of 10,000 reads. In addition to Coral, we compared the performance of DUDE-Seq with that of BLESS [48], fermi [49], and Trowel [25], which are representative *k*-mer-based state-of-the-art tools. BLESS corrects “weak” *k*-mers that exist between consecutive “solid” *k*-mers, assuming that a weak *k*-mer has only one error. Fermi corrects sequencing errors in underrepresented *k*-mers using a heuristic cost function based on quality scores and does not rely on a *k*-mer occurrence threshold. Trowel does not use a coverage threshold for its *k*-mer spectrum and iteratively boosts the quality values of bases after making corrections with *k*-mers that have high quality values.

**Table 1:** Details of the Illumina datasets [32] used for our experiments shown in Fig 7.

dataset ID	region	sequencer	Taq	organism	forward & reverse primer
Q19	V4	MiSeq2	Q5	AT	515 & 805RA
Q20	V4	MiSeq2	Q5	BT	515 & 805RA
Q21	V4	MiSeq2	Q5	BV	515 & 805RA
Q22	V4	MiSeq2	Q5	CS	515 & 805RA
Q23	V4	MiSeq2	HF	AT	515 & 805RA
Q24	V4	MiSeq2	HF	BT	515 & 805RA
Q25	V4	MiSeq2	HF	BV	515 & 805RA
Q26	V4	MiSeq2	HF	CS	515 & 805RA
Q27	V3/V4	MiSeq1	Q5	AT	314f & 806rcb
Q28	V3/V4	MiSeq1	Q5	BT	314f & 806rcb
Q29	V3/V4	MiSeq1	Q5	BV	314f & 806rcb
Q30	V3/V4	MiSeq1	Q5	CS	314f & 806rcb
Q31	V3/V4	MiSeq1	HF	AT	314f & 806rcb

Taqs: HiFI Kapa (HF), Q5 neb (Q5); Organisms: Anaerocellum thermophilum Z-1320 DSM 6725 (AT), Bacteroides thetaiotaomicron VPI-5482 (BT), Bacteroides vulgatus ATCC 8482 (BV), Caldicellulosiruptor saccharolyticus DSM 8903 (CS), Herpetosiphon aurantiacus ATCC 23779 (HA), Rhodopirellula baltica SH 1 (RBS), Leptothrix cholodnii SP-6 (LC)

Fig 7 shows the per-base error rates, defined in Eq (6), for the tools under comparison using the first eight datasets (Q19–Q26) and their subsets created as described above (thus, a total of 80 datasets per tool). BLESS did not run successfully on these datasets, and hence its results are not shown. First, we can confirm that DUDE-Seq is effective in reducing substitution errors for data obtained using the Illumina platform in all tested cases of targeted amplicon sequencing, with relative error rate reductions of 6.40–49.92%, compared to the ‘Raw’ sequences. Furthermore, among the tools included in the comparison, DUDE-Seq produced the best results for the largest number of datasets. For Q24 and Q25, fermi was most effective, but was outperformed by DUDE-Seq in many other cases. Coral was able to denoise to some extent but was inferior to DUDE-Seq and fermi. Trowel gave unsatisfactory results in this experiment.

Before presenting our next results, we note that while the error rate defined in Eq (6) is widely used for DNA sequence denoising research as a performance measure, it occasionally misleading and cannot be used to fairly evaluate the performance of denoisers. This is because only errors at aligned bases are counted in the error rate calculation; hence, a poor denoiser may significantly reduce the number of aligned bases, potentially further corrupting the noisy sequence, but it can have a low error rate calculated as in Eq (6). In our experiments with the datasets Q27-Q31, we detected a large variance in the number of aligned bases across different denoising tools; thus, it was difficult to make a fair comparison among the performance of different tools with Eq (6). We note that in the experiments presented in Figs 6(a) and 7, such a large variance was not detected. To alleviate this issue, we employ the alternative definition of the per-base error rate of a tool in Eq (8).

**Fig 8:**
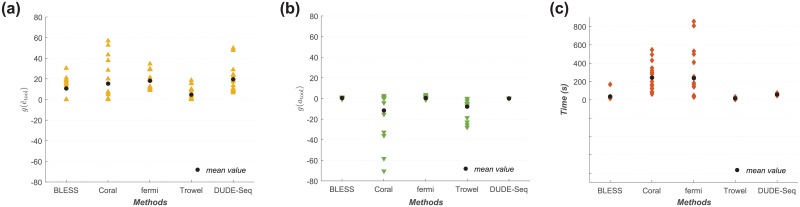
Performance comparison. (a) Relative gain of adjusted error rates over ‘Raw’ data Eq (9). (b) Relative gain of aligned bases Eq (7). (c) Running time on real Illumina datasets (labeled Q19–Q31; see the caption for Fig 7). [parameters: kmerlength = 21 for BLESS; (*k*, *mr*, *mm*, *g*) = (21, 1, 1, 1000) for Coral; *k* = 21 for Trowel; (*k*, *O*, *C*, *s*) = (21, 3, 0.3, 5) for fermi; *k* = 5 for DUDE-Seq] [BLESS did not work on Q19–Q26].

Fig 8 shows the results obtained for 100,000-read subsets of each of the Q19–Q31 datasets, i.e., all datasets, for DUDE-Seq and the four alternative denoisers. Because the datasets Q27–Q31 had two subsets of 100,000 reads, we used a total of 18 datasets to draw Fig 8, one each from Q19–Q26 and two each from Q27–Q31. As mentioned previously, BLESS could not run successfully on Q19–Q26; hence, there are only 10 points for BLESS in the plots. Fig 8(a), 8(b) and 8(c) presents the distribution of $g({\hat{e}}_{\text{tool}})$, *g*(*a*_tool_), and running times for each tool, respectively. For each distribution, the average value is marked with a solid circle. As shown in Fig 8(b), we clearly see that Coral and Trowel show a large variance in the number of aligned bases. For example, Coral only aligns 30% of bases compared to the raw sequence after denoising for some datasets. With the effect of this variance in aligned bases adjusted, Fig 8(a) shows that DUDE-Seq produces the highest average $g({\hat{e}}_{\text{tool}})$, *i.e.*, 19.79%, among all the compared tools. Furthermore, the variability of *g*(*a*_tool_) was the smallest for DUDE-Seq, as shown in Fig 8(b), suggesting its robustness. Finally, in Fig 8(c), we observe that the running times were significantly shorter for DUDE-Seq and Trowel than for Coral and fermi. Overall, we can conclude that DUDE-Seq is the most robust tool, with a fast running time and the highest average accuracy after denoising.

In summary, we observe from Figs 7 and 8 that DUDE-Seq robustly outperforms the competing schemes for most of the datasets tested. We specifically emphasize that DUDE-Seq shows a strong performance, even though the DMC assumption does not hold for the sequencer. We believe that the better performance of DUDE-Seq relative to other state-of-the-art algorithms (based on MSA or *k*-mer spectrums) on real Illumina datasets strongly demonstrates the competitiveness of DUDE-Seq as a general DNA sequence denoiser for targeted amplicon sequencing.

### Experiments on simulated data

We performed more detailed experiments using Illumina simulators in order to further highlight the strong denoising performance of DUDE-Seq, including the effects on downstream analyses.

**Fig 9:**
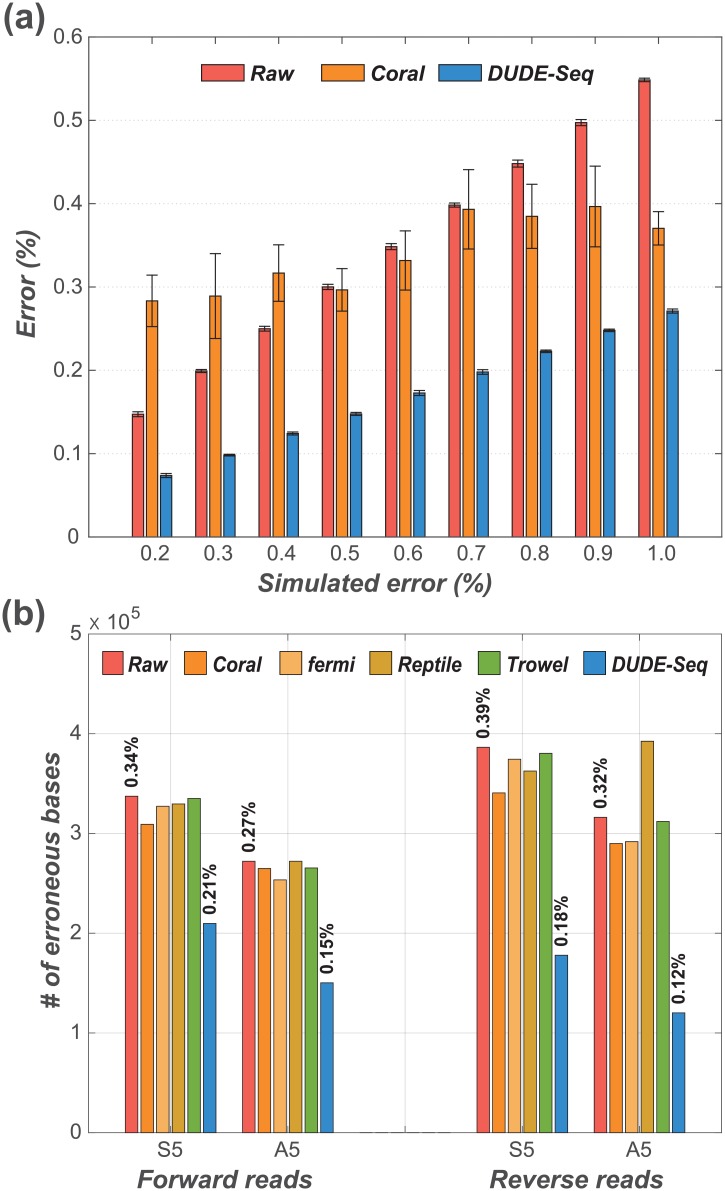
Reads correction performance on simulated dataset. [parameters: *k* = 5 for DUDE-Seq; *k* = 10 for Trowel; (*k*, *mr*, *mm*, *g*) = (21, 1, 1, 1000) for Coral; optimal values set by tool seq-analy for Reptile; (*k*, *O*, *C*, *s*) = (21, 3, 0.3, 5) for fermi]: (a) Varying error rates using the Grinder simulator [50]. (b) Varying reads composition using the GemSIM simulator [51] (values on top of each bar represent the error rates).

Fig 9(a) shows the results obtained using the Grinder simulator [50] and a comparison with Coral. Trowel and Reptile require quality scores as input, which are provided by the GemSIM simulator, but not by the Grinder simulator; hence, we could not include Trowel and Reptile in Fig 9(a). We generated nine synthetic datasets of forward reads that had error rates at the end of the sequence varying from 0.2% to 1.0%, as denoted on the horizontal axis. For all cases, the error rate at the beginning of the sequence was 0.1%. We again used the *average* DMC model for the entire sequence for DUDE-Seq. Note that the error rates for the ‘Raw’ data, *i.e.*, the red bars, match the average of the error rates at the beginning and the end of the sequence. From the figure, consistent with the real datasets analyzed in Section, we clearly see that DUDE-Seq significantly outperforms Coral for all tested error rates.

To evaluate the performance of DUDE-Seq for paired-end reads, we generated datasets, shown in Table 2, with the GemSIM sequencing data simulator [51]. As shown in the table, we used 23 public reference sequences [35] to generate the dataset A5 and a single reference sequence for S5. We used the error model v5 that has error rate s of 0.28% for forward reads and 0.34% for reverse reads. In Fig 9(b), in addition to DUDE-Seq, Coral, fermi, and Trowel, we included the results obtained using Reptile [20], another *k*-mer spectrum-based method that outputs read-by-read denoising results. We again observe from the figure that DUDE-Seq outperforms the alternatives by significant margins.

**Table 2:** Details of the public data [52] used for our experiments on simulated data shown in Table 3.

dataset ID	# total reads	# refs	fragment length	read length	overlap length	simulator (error model) or sequencer used
S5	1,000,000	[1]	160	100	40	GemSIM (v5^#^)
A5	1,000,000	[23]	160–190	100	10–40	GemSIM (v5^^#^^)

^*#*^ Error model v5 (forward rate 0.28%, reverse 0.34%)

In Table 3, we show that the error-corrected reads produced by DUDE-Seq can also improve the performance of downstream pipelines, such as paired-end merging. We applied four different paired-end merging schemes, CASPER [52], COPE [53], FLASH [47], and PANDAseq [54], for the two datasets A5 and S5 in Table 2. The metrics are defined as usual. A true positive (TP) is defined as a merge with correct mismatching resolution in the overlap region, and a false positive (FP) is defined as a merge with incorrect mismatching resolution in the overlap region. Furthermore, a false negative (FN) is a merge that escapes the detection, and a true negative (TN) is defined as a correct prediction for reads that do not truly overlap. The accuracy and F1 score are computed based on the above metrics [55]. For each dataset, we compared the results for four cases: performing paired-end merging without any denoising, after correcting errors with Coral, after correcting errors with fermi, and after correcting errors with DUDE-Seq. Reptile and Trowel were not included in this experiment because they were generally outperformed by Coral and fermi, as shown in Fig 9(b). The accuracy and F1 score results show that correcting errors with DUDE-Seq consistently yields better paired-end merging performance, not only compared to the case with no denoising, but also compared to the cases with Coral and fermi error correct ion. This result highlights the potential application of DUDE-Seq for boosting the performance of downstream DNA sequence analyses.

**Table 3:** Paired-end reads merging performance statistics [parameters: *k* = 5 for DUDE-Seq; (*k*, *mr*, *mm*, *g*) = (21, 1, 1, 1000) for Coral; (*k*, *O*, *C*, *s*) = (21, 3, 0.3, 5) for fermi].

tool	dataset	# merges	TP	FP	FN	accuracy	*F*_1_
CASPER	S5	1,000,000	997,303	2,697	**0**	0.997	0.999
COPE		974,219	961,366	12,853	25,781	0.961	0.980
FLASH		999,921	977,431	22,490	79	0.977	0.989
PANDAseq		999,947	976,807	23,140	53	0.977	0.988
CASPER	S5 w/ Coral	1,000,000	997,510	2,490	**0**	0.998	0.999
COPE		975,803	963,717	12,086	24,197	0.964	0.982
FLASH		999,942	978,835	21,107	58	0.979	0.989
PANDAseq		999,949	978,270	21,679	51	0.978	0.989
CASPER	S5 w/ fermi	1,000,000	997,356	2,644	**0**	0.997	0.999
COPE		994,025	969,451	24,574	**5,975**	0.969	0.984
FLASH		999,933	972,025	27,908	67	0.972	0.986
PANDAseq		999,952	971,567	28,385	48	0.972	0.986
CASPER	S5 w/ DUDE-Seq	1,000,000	**999,320**	**680**	**0**	**0.999**	**1.000**
COPE		987,238	**983,639**	**3,599**	12,762	**0.984**	**0.992**
FLASH		999,958	**992,915**	**7,043**	**42**	**0.993**	**0.996**
PANDAseq		999,949	**991,146**	**8,803**	**51**	**0.991**	**0.996**
CASPER	A5	999,973	997,202	2,771	27	0.997	**0.999**
COPE		924,634	915,981	8,653	75,366	0.916	0.956
FLASH		999,578	977,355	22,223	422	0.977	0.989
PANDAseq		999,122	978,720	20,402	878	0.979	0.989
CASPER	A5 w/ Coral	999,974	995,899	4,075	**26**	0.996	0.998
COPE		927,757	918,733	9,024	72,243	0.919	0.958
FLASH		999,742	978,814	20,928	**258**	0.979	0.989
PANDAseq		999,351	979,899	19,452	649	0.980	0.990
CASPER	A5 w/ fermi	999,969	997,288	2,681	31	0.997	**0.999**
COPE		939,986	923,252	16,734	60,014	0.923	0.960
FLASH		999,732	974,903	24,829	268	0.975	0.987
PANDAseq		999,328	975,320	24,008	672	0.975	0.988
CASPER	A5 w/ DUDE-Seq	999,971	**998,078**	**1,893**	29	**0.998**	**0.999**
COPE		943,531	**939,555**	**3,976**	**56,469**	**0.940**	**0.969**
FLASH		999,638	**989,860**	**9,778**	362	**0.990**	**0.995**
PANDAseq		999,354	**989,250**	**10,104**	**646**	**0.989**	**0.995**

## Discussion

Our experimental results show that DUDE-Seq can robustly outperform *k*-mer-based, MSA-based, and statistical error model-based schemes for both real-world datasets, such as 454 pyrosequencing and Illumina data, and simulated datasets, particularly for targeted amplicon sequencing. This performance advantage in denoising further allowed us to obtain improved results in downstream analysis tasks, such as OTU binning and paired-end merging. Furthermore, the time demand of DUDE-Seq-based OTU binning is order(s) of magnitude lower than that of the current state-of-the-art schemes. We also demonstrated the robustness and flexibility of DUDE-Seq by showing that a simple change in **Π** matrix is enough to apply the exact same DUDE-Seq to data obtained using different sequencing platforms. In particular, we experimentally showed that even when the memoryless channel assumption does not hold, as in Illumina data, DUDE-Seq still solidly outperforms state-of-the-art schemes.

The sliding window nature of DUDE-Seq resemble s the popular *k*-mer-based schemes in the literature. However, while all existing *k*-mer-based schemes rely on heuristic threshold selection for determining errors in the reads, regardless of the error model of the sequencing platform, DUDE-Seq applies an analytic denoising rule that explicitly takes the error model **Π** into account. Therefore, even for identical noisy reads *z*^*n*^, DUDE-Seq may result in different denoised sequences, depending on the **Π**’s of different sequencing platforms, whereas the *k*-mer-based scheme will always result in the exact same denoised sequence. The performance gains reported in this paper compared to state-of-the-art baselines, including those for *k*-mer-based schemes, substantiate the competitiveness of our method for targeted amplicon sequencing.

Another advantage of DUDE-Seq is its read-by-read error-correction capability. This feature is important for a number of bioinformatics tasks, including *de novo* sequencing, metagenomics, resequencing, targeted resequencing, and transcriptome sequencing, which typically require the extraction of subtle information from small variants in each read. In addition to the types of tasks presented in this paper (*i.e.*, per-based error correction, OTU binning, and paired-end merging), we plan to apply DUDE-Seq to additional tasks, as mentioned above.

Additional venues for further investigation include the procedure for estimating the noise mechanism represented by **Π**, which is currently empirically determined by aligning each read to the reference sequence and is therefore sensitive to read mapping and alignment. For more robust estimation, we may employ an expectation-maximization-based algorithm, as was recently proposed for estimating substitution emissions for the data obtained using nanopore technology [56]. Considering uncertainties in **Π** may also be helpful; hence, it may be useful to investigate the relevance of the framework in [57]. Additionally, it will likely be fruitful to utilize the information in Phred quality scores to make decisions about noisy bases and to fine-tune the objective loss function in our approach. Using a lossy compressed version of the quality scores is one possible direction for boosting the inferential performance of some downstream applications, as shown in [58]. Furthermore, particularly for the homopolymer error correction, there are several hyperparameters whose choices can be experimented with in the future to potentially achieve substantial performance boosts. Examples include the choice of alphabet size (in lieu of the current value of 10), the choice of the loss function that may be proportional to the difference between the true and estimated value of *N* (in lieu of the current Hamming loss), and the choice of quantization (in lieu of Eq (4)). Moreover, we may apply the full generalized DUDE in [34] for homopolymer error correction to determine if better performance can be achieved at the cost of increased complexity. Applying DUDE-Seq to other types of sequencing technology with homopolymer errors (*e.g.*, Ion Torrent) would also be possible as long as we can acquire flow (*e.g.*, ionogram) density distributions to estimate **Γ**. Currently, there exists no public data repository that includes such information for Ion Torrent, and thus existing Ion Torrent denoisers often ignore homopolymer errors or rely on simplistic noise modeling and iterative updates that unrealistically limit the maximum length of homopolymer errors that can be handled, let alone computational efficiency [36]. Finally, we plan to test DUDE-Seq on several other sequencing platforms, such as PacBio and Oxford Nanopore, which tend to result in longer and more noisy sequences, to further substantiate the robustness and effectiveness of our algorithm. Applying the recently developed deep neural networks -based Neural DUDE algorithm [59] to DNA sequence denoising beyond targeted amplicon sequencing could be another fruitful direction.

## Supporting information

S1 File**Fig A, DUDE-Seq web interface.** This is a screenshot of the website accompanying the proposed DUDE-Seq method (http://data.snu.ac.kr/pub/dude-seq). For users who prefer a graphical user interface, this website provides a web-based execution environments for DUDE-Seq. Through this screen, a user can specify the parameters for each of the two error types (in the figure, DUDE-Seq (1) stands for for the substitution error correction described in Algorithm 1 and DUDE-Seq (2) stands for the homopolymer error correction shown in Algorithm 2), and upload the input file of her choice. The DUDE-Seq process starts automatically by clicking the “SUBMIT” button. For advanced users who prefer batch processing, the source code of DUDE-Seq is also available at http://github.com/datasnu/dude-seq. All the used datasets are also available on the Sequence Read Archive (SRA) under the accession number SRP000570 (SRS002051–SRS002053) at https://www.ncbi.nlm.nih.gov/sra/SRP000570 and the European Nucleotide Archive (ENA) under the accession number PRJEB6244 (ERS671332–ERS671344) at http://www.ebi.ac.uk/ena/data/view/PRJEB6244. **Fig B, Website output: sequence complexity**. The DUDE-Seq website provides analysis results from applying the DUST algorithm [60] and block-entropy to the outputs from denoising by DUDE-Seq. The DUST algorithm masks low-complexity regions that have highly biased distribution of nucleotides based on counting 3-mer frequencies in 64-base windows. The DUST score is computed based on how often different trinucleotides occur as follows:
$$\text{score}=\sum_{i=1}^{k}\frac{n_{i}(n_{i}-1)(w-2)s}{2(l-1)l}$$
where *k* = 4^3^ is the trinucleotide size, *w* = 64 is the window size, *n*_*i*_ is the number of the words *i* in a window, *l* is the number of the possible words in a window, and *s* is the scaling factor. The score is scaled from 0 to 100 and a high score implies a low complexity metagenome. The block-entropy is calculated using Shannon’s diversity index [61]. The block-entropy evaluates the entropy of the trinucleotides in a sequence as follows:
$$\text{entropy}=-\sum_{i=1}^{k}(\frac{n_{i}}{l}){\text{log}}_{k}(\frac{n_{i}}{l})$$
where *k* = 4^3^ is the trinucleotide size, *n*_*i*_ is the number of the words *i* in a window, and *l* is the number of the possible words in a window. The entropy is also scaled from 0 to 100 and a low entropy implies a low complexity metagenome. **Fig C, Website output: tag sequence probability**. Another output from the DUDE-Seq website is the tag sequence probability of reads [62]. This is to reveal the existence of artifacts at the ends, *i.e.*, adapter or barcode sequences at the 5’- or 3’-end. **Fig D, Website output: sequence duplication**. The accompanying website also carries out sequence duplication analysis based on the denoised outputs from DUDE-Seq, in order to reveal artificial duplicates. As shown in the figure, five types of duplication statistics [63] are provided: exact duplicates, 5’ duplicates, 3’ duplicates, exact duplicates with the reverse complement of another sequence, and 5’ or 3’ duplicates with the reverse complement of another sequence.(PDF)
