# Supplementary material for: DUDE-Seq: Fast, flexible, and robust denoising for targeted amplicon sequencing
Source: PLoS One. 2017 Jul 27;12(7):e0181463. doi: 10.1371/journal.pone.0181463 (PMC5531809; doi:10.1371/journal.pone.0181463)
Supplement: S1 File — Fig A, DUDE-Seq web interface. This is a screenshot of the website accompanying the proposed DUDE-Seq method (http://data.snu.ac.kr/pub/dude-seq). For users who prefer a graphical user interface, this website provides a web-based execution environments for DUDE-Seq. Through this screen, a user can specify the parameters for each of the two error types (in the figure, DUDE-Seq (1) stands for for the substitution error correction described in Algorithm 1 and DUDE-Seq (2) stands for the homopolymer error correction shown in Algorithm 2), and upload the input file of her choice. The DUDE-Seq process starts automatically by clicking the “SUBMIT” button. For advanced users who prefer batch processing, the source code of DUDE-Seq is also available at http://github.com/datasnu/dude-seq. All the used datasets are also available on the Sequence Read Archive (SRA) under the accession number SRP000570 (SRS002051–SRS002053) at https://www.ncbi.nlm.nih.gov/sra/SRP000570 and the European Nucleotide Archive (ENA) under the accession number PRJEB6244 (ERS671332–ERS671344) at http://www.ebi.ac.uk/ena/data/view/PRJEB6244. Fig B, Website output: sequence complexity. The DUDE-Seq website provides analysis results from applying the DUST algorithm [60] and block-entropy to the outputs from denoising by DUDE-Seq. The DUST algorithm masks low-complexity regions that have highly biased distribution of nucleotides based on counting 3-mer frequencies in 64-base windows. The DUST score is computed based on how often different trinucleotides occur as follows: score=∑i=1kni(ni−1)(w−2)s2(l−1)l where k = 43 is the trinucleotide size, w = 64 is the window size, ni is the number of the words i in a window, l is the number of the possible words in a window, and s is the scaling factor. The score is scaled from 0 to 100 and a high score implies a low complexity metagenome. The block-entropy is calculated using Shannon’s diversity index [61]. The block-entropy evaluates the entropy of the trinucleotides [file pone.0181463.s001.pdf]

[Supporting Information]

DUDE-Seq: Fast, Flexible, and Robust Denoising for Targeted Amplicon Sequencing

Byunghan Lee[1], Taesup Moon[2*], Sungroh Yoon[1,3,4*], and Tsachy Weissman[5]

[1] *Electrical and Computer Engineering, Seoul National University, Seoul, Korea*
[2] *College of Information and Communication Engineering, Sungkyunkwan University, Suwon, Korea*
[3] *Interdisciplinary Program in Bioinformatics, Seoul National University, Seoul, Korea*
[4] *Neurology and Neurological Sciences, Stanford University, Stanford, California, United States of America*
[5] *Department of Electrical Engineering, Stanford University, Stanford, California, United States of America*

Source Code Availability and DUDE-Seq Website

The website accompanying DUDE-Seq is available at http://data.snu.ac.kr/pub/dude-seq), as shown in Fig. A. For users who prefer a graphical user interface, this website provides a web-based execution environments for DUDE-Seq. Through this screen, a user can specify the parameters for each of the two error types and upload the input file of her choice. The users can upload a `.zip` file which contains single `.dat` [1] or `.fasta` or `.fastq` file for analysis. If the submitted data is `.fasta` or `.fastq` file, we proceed Algorithm 1 alone, otherwise, do Algorithm 2 followed by Algorithm 1. For advanced users who prefer batch processing, the source code of DUDE-Seq is also available at http://github.com/datasnu/dude-seq.

All the used datasets are also available on the Sequence Read Archive (SRA) under the accession number SRP000570 (SRS002051–SRS002053) at https://www.ncbi.nlm.nih.gov/sra/SRP000570 and the European Nucleotide Archive (ENA) under the accession number PRJEB6244 (ERS671332–ERS671344) at http://www.ebi.ac.uk/ena/data/view/PRJEB6244.

In addition to denoising by DUDE-Seq, the website provides additional analysis of the sequencing results that should be useful for the downstream analyses. The DUDE-Seq website utilizes PRINSEQ [2] to provide the following statistics to examine and control the quality of sequencing data: the length distribution, the `GC` content distribution, the occurrence of the ambiguous base `N`s, the tag sequence probability, the sequence duplication, and the sequence complexity.

Length distribution lists the mean, minimum, maximum, and mode sequence lengths. **`GC` content distribution** lists the mean, minimum, maximum, and mode `GC` contents. **Sequence complexity** shown in Fig. B is calculated using the DUST algorithm [3] and block-entropy. The DUST algorithm masks low-complexity regions that have highly biased distribution of nucleotides based on counting 3-mer frequencies in 64-base windows. The block-entropy is calculated using Shannon's diversity index [4]. **Tag sequence probability** shown in Fig. C is calculated to reveal the existence of artifacts at the ends, *i.e.*, adapter or barcode sequences at the 5'- or 3'-end, according to Schmieder *et al.* [5]. **Sequence duplication** shown in Fig. D is calculated to relieve the artificial

*To whom correspondence should be addressed: tsmoon@skku.edu (TM), sryoon@snu.ac.kr (SY)

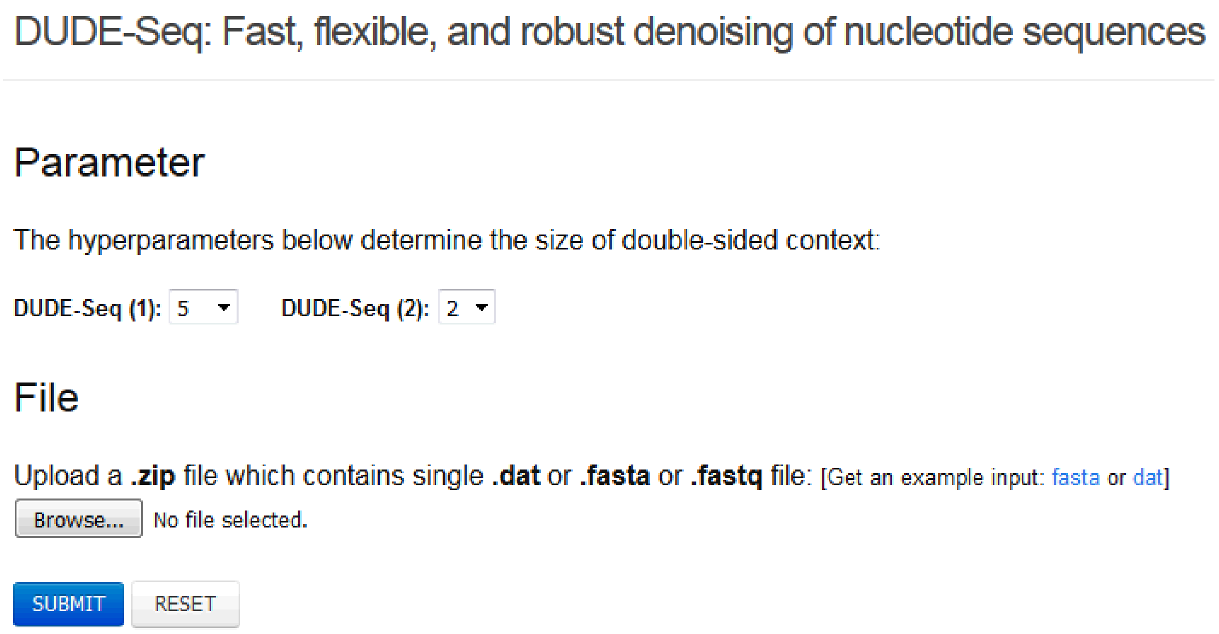

Fig. A: **DUDE-Seq web interface.** This is a screenshot of the website accompanying the proposed DUDE-Seq method (http://data.snu.ac.kr/pub/dude-seq). For users who prefer a graphical user interface, this website provides a web-based execution environments for DUDE-Seq. Through this screen, a user can specify the parameters for each of the two error types (in the figure, DUDE-Seq (1) stands for for the substitution error correction described in Algorithm 1 and DUDE-Seq (2) stands for the homopolymer error correction shown in Algorithm 2), and upload the input file of her choice. The DUDE-Seq process starts automatically by clicking the "SUBMIT" button. For advanced users who prefer batch processing, the source code of DUDE-Seq is also available at http://github.com/datasnu/dude-seq.

duplicates. The duplicates are categorized into five groups [2]: exact duplicates, 5' duplicates, 3' duplicates, exact duplicates with the reverse complement of another sequence, and 5' or 3' duplicates with the reverse complement of another sequence.

References

[1] C. Quince, A. Lanzén, T. P. Curtis, R. J. Davenport, N. Hall, I. M. Head, L. F. Read, and W. T. Sloan, "Accurate determination of microbial diversity from 454 pyrosequencing data," *Nature methods*, vol. 6, no. 9, p. 639, 2009.

[2] R. Schmieder and R. Edwards, "Quality control and preprocessing of metagenomic datasets," *Bioinformatics*, vol. 27, no. 6, pp. 863–864, 2011.

[3] A. Morgulis, E. M. Gertz, A. A. Schäffer, and R. Agarwala, "A fast and symmetric dust implementation to mask low-complexity dna sequences," *Journal of Computational Biology*, vol. 13, no. 5, pp. 1028–1040, 2006.

[4] C. E. Shannon, "A mathematical theory of communication," *ACM SIGMOBILE Mobile Computing and Communications Review*, vol. 5, no. 1, pp. 3–55, 2001.

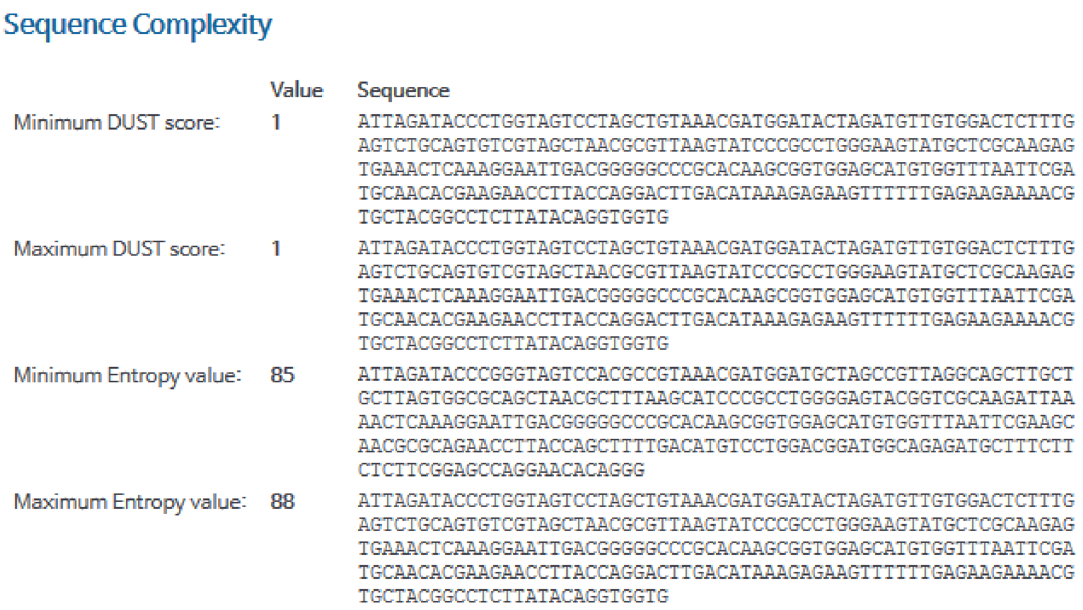

Sequence Complexity

	Value	Sequence
Minimum DUST score:	1	ATTAGATACCCTGGTAGTCCTAGCTGTAAACGATGGATACTAGATGTTGTGGACTCTTTG<br>AGTCTGCAGTGTCGTAGCTAACGCGTTAAGTATCCCGCCTGGGAAGTATGCTCGCAAGAG<br>TGAAACTCAAAGGAATTGACGGGGGCCCGCACAAGCGGTGGAGCATGTGGTTTAATTCGA<br>TGCAACACGAAGAACCTTACCAGGACTTGACATAAAGAGAAGTTTTTTGAGAAGAAAACG<br>TGCTACGGCCTCTTATACAGGTGGTG
Maximum DUST score:	1	ATTAGATACCCTGGTAGTCCTAGCTGTAAACGATGGATACTAGATGTTGTGGACTCTTTG<br>AGTCTGCAGTGTCGTAGCTAACGCGTTAAGTATCCCGCCTGGGAAGTATGCTCGCAAGAG<br>TGAAACTCAAAGGAATTGACGGGGGCCCGCACAAGCGGTGGAGCATGTGGTTTAATTCGA<br>TGCAACACGAAGAACCTTACCAGGACTTGACATAAAGAGAAGTTTTTTGAGAAGAAAACG<br>TGCTACGGCCTCTTATACAGGTGGTG
Minimum Entropy value:	85	ATTAGATACCCGGGTAGTCCACGCCGTAAACGATGGATGCTAGCCGTTAGGCAGCTTGCT<br>GCTTAGTGGCGCAGCTAACGCTTTAAGCATCCCGCCTGGGGAGTACGGTCGCAAGATTAA<br>AACTCAAAGGAATTGACGGGGGCCCGCACAAGCGGTGGAGCATGTGGTTTAATTCGAAGC<br>AACGCGCAGAACCTTACCAGCTTTTGACATGTCCTGGACGGATGGCAGAGATGCTTTCTT<br>CTCTTCGGAGCCAGGAACACAGGG
Maximum Entropy value:	88	ATTAGATACCCTGGTAGTCCTAGCTGTAAACGATGGATACTAGATGTTGTGGACTCTTTG<br>AGTCTGCAGTGTCGTAGCTAACGCGTTAAGTATCCCGCCTGGGAAGTATGCTCGCAAGAG<br>TGAAACTCAAAGGAATTGACGGGGGCCCGCACAAGCGGTGGAGCATGTGGTTTAATTCGA<br>TGCAACACGAAGAACCTTACCAGGACTTGACATAAAGAGAAGTTTTTTGAGAAGAAAACG<br>TGCTACGGCCTCTTATACAGGTGGTG

Fig. B: **Website output: sequence complexity.** The DUDE-Seq website provides analysis results from applying the DUST algorithm [3] and block-entropy to the outputs from denoising by DUDE-Seq. The DUST algorithm masks low-complexity regions that have highly biased distribution of nucleotides based on counting 3-mer frequencies in 64-base windows. The block-entropy is calculated using Shannon's diversity index [4].

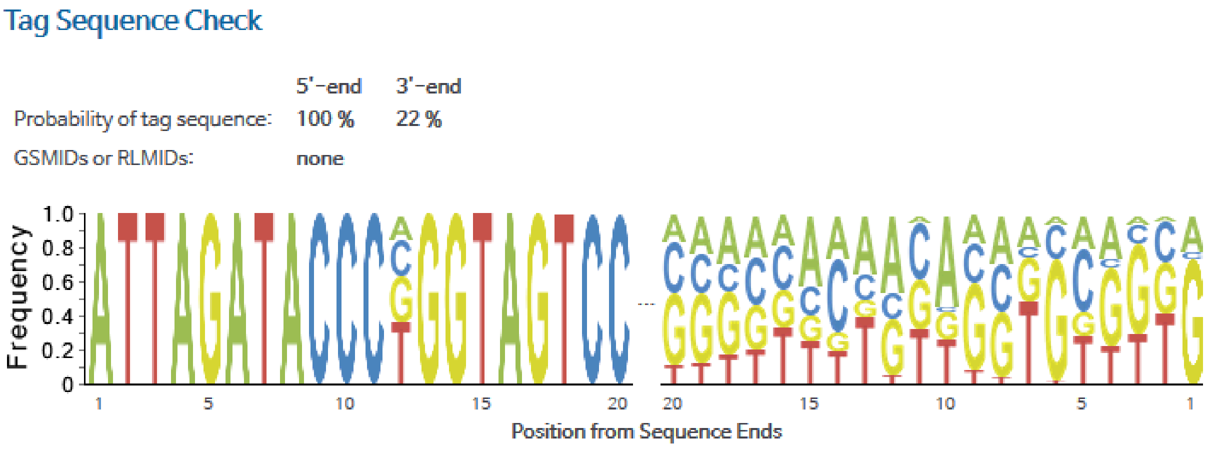

Fig. C: **Website output: tag sequence probability.** Another output from the DUDE-Seq website is the tag sequence probability of reads [5]. This is to reveal the existence of artifacts at the ends, *i.e.*, adapter or barcode sequences at the 5'- or 3'-end.

Sequence Duplication

	# Sequences	Max duplicates
Exact duplicates:	26,326 (82.61 %)	1422
Exact duplicates with reverse complements:	0	0
5' duplicates	975 (3.06 %)	4
3' duplicates	0	0
5'/3' duplicates with reverse complements	0	0
Total:	27,301 (85.67 %)	-

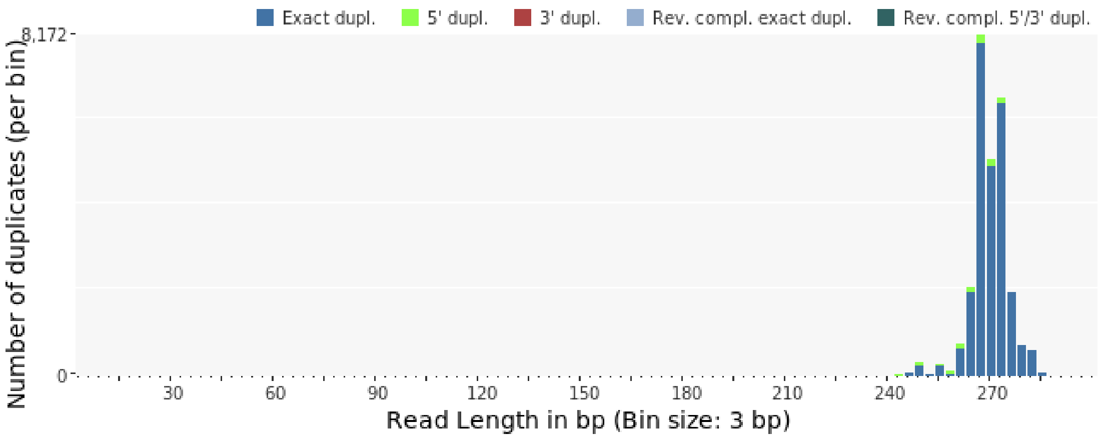

Fig. D: **Website output: sequence duplication.** The accompanying website also carries out sequence duplication analysis based on the denoised outputs from DUDE-Seq, in order to reveal artificial duplicates. As shown in the figure, five types of duplication statistics [2] are provided: exact duplicates, 5' duplicates, 3' duplicates, exact duplicates with the reverse complement of another sequence, and 5' or 3' duplicates with the reverse complement of another sequence.

[5] R. Schmieder, Y. W. Lim, F. Rohwer, and R. Edwards, “Tagcleaner: Identification and removal of tag sequences from genomic and metagenomic datasets,” *BMC bioinformatics*, vol. 11, no. 1, p. 1, 2010.
